# Investigation of Mechanical Properties of Silicone/Phosphor Composite Used in Light Emitting Diodes Package

**DOI:** 10.3390/polym10020195

**Published:** 2018-02-15

**Authors:** Yongjun Pan, Fulong Zhu, Jiajie Fan, Jiaquan Tao, Xinxin Lin, Fengren Wang, Lang Shi

**Affiliations:** 1 Institute of Microsystems, School of Mechanical Science and Engineering, Huazhong University of Science and Technology, 1037 Luoyu Road, Wuhan 430074, China; yongjun_pan@hust.edu.cn (Y.P.); 15527008850@163.com (J.T.); linxinxin@hust.edu.cn (X.L.); m201670358@hust.edu.cn (F.W.); m201670357@hust.edu.cn (L.S.); 2 School of Mechanical and Electrical Engineering, Hohai University, 200 Jinling Bei Road, Changzhou 213022, China; jay.fan@connect.polyu.hk

**Keywords:** mechanical properties, aging, CZM, interface debonding

## Abstract

Mass fraction of phosphor in silicone and aging time play important roles in the optics and mechanical performance of the silicone that is used in the light emitting diode (LED) package. In this paper, the mechanical properties of silicone/phosphor composites are investigated experimentally by separate tensile and compression tests. Distribution of the phosphors is observed by scanning electron microscopy (SEM) to ensure the homogeneity of the samples. Different loading rates are applied to study the silicone material’s rate-dependent properties. The experimental results of the tensile and compression test show that the Young’s modulus increases with the mass fraction of phosphor in silicone. Longer aging time stiffens the silicone composite and weakens the ductility of the materials. A three-dimensional model used cohesive zone material (CZM) between the interface of the phosphor particles, and matrix silicone is built up to study the degradation mechanism at a micro-scale level. The simulation results indicate that the diameter of particles in silicone also impacts its interface debonding and crack growth. The theoretical results concerning the mass fraction of phosphor are in good agreement with the experiments.

## 1. Introduction

Their high efficiency and reliability led white light-emitting diodes (LEDs) to become an indispensable solid-state light source for indoor and outdoor illumination [1,2,3,4]. There are several components used in LED packaging, but the component that has the greatest influence is the color-converting material [5]. Colloidal semiconductor nanocrystals (quantum dots) have widely been used for color-converting materials globally. Combining the properties of the polymer matrix and the quantum dots could lead to high-performance composite materials that are needed for the LED package [6,7]. Owing to the transparency of silicone, it has been widely used as a polymer matrix. According to the work by Zhaohui Chen et al. [8,9,10,11], it was found that the mechanical properties of silicone composite have a great influence on the life time of LED products. Due to the high coefficient of thermal expansion, the bonding wire embedded in the composite could experience severe stress conditions, and could finally crack. In our reliability test, the samples with different mass fractions of phosphor had different lifetimes before the occurrence of failure. Therefore, the mass fraction of phosphor plays an important role in the mechanical properties of the composite. It’s essential to study the mechanical properties of this composite. Xing Chen et al. [12] investigated different volume fractions of silicone/phosphor composites by tensile experiment and simulation. It’s much easier to control the mass fraction instead of the volume fraction during the preparation of samples.

In LED products, bonding wires, which are used for electrical interconnection, are embedded in the silicone/phosphor composite. During thermal cycle testing, the composite shrinks and expands with temperature change. This change could cause fatigue cracking in bonding wire. In order to study the mechanical response of the composite during thermal cycling, load/unload tests are a necessary part of the experiment. In this paper, the mechanical behaviors of silicone/phosphor composites are obtained by both compression and tensile testing. The finite element method is adopted to obtain the stress and strain distribution in composite. The silicone matrix is simulated with the neo-Hookean hyperelastic material model [13,14]. The cohesive zone material model is used to simulate the cohesive strength of the interface between silicone and phosphor. The results obtained by simulation are validated by experimental data.

## 2. Experiment Preparation

To prepare the samples used in the experiments, Ce^3+^-doped Y_3_Al_5_O_12_ (Ce: YAG) power was adopted as the phosphor filler. Table 1 lists the basic mechanical properties of silicone and phosphor [12]. The Young’s modulus of silicone is obtained by compression test of the pure silicone. Nanoindentation is used to obtain the Young’s modulus of phosphor. The mass fraction of phosphor used in LED products is determined based on the volume of phosphor particles, usually between 10% and 20%. To investigate the impact of the mass fraction of phosphor, four groups of samples are prepared for compression tests, one is pure silicone, and the others contain different mass fractions of phosphor, which are controlled at 10%, 20% and 40%.

**Figure 1:**
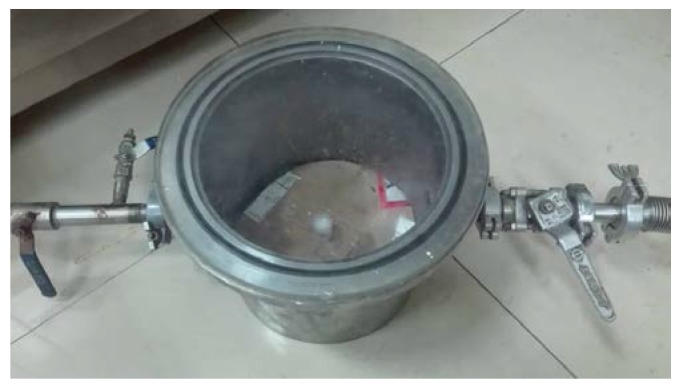
Vacuum tank used for eliminating air bubbles.

There were 3 main steps to fabricating the samples: (1) Component A and component B were mixed in equal volumes in a breaker. Phosphor filler was added into the mixture in a certain amount, which was controlled by electronic balance (Liang Ping Instruments, Shanghai, China). Then the mixture was stirred for about 15 min by dispersing homogenizer (OuHor, Shanghai, China). The homogenizer’s spin velocity was set at 300 rpm to mix the silicone and phosphor perfectly. (2) The vacuum tank (Wuhan National Laboratory for Optoelectronics, Wuhan, China) shown in Figure 1 was employed to exclude air bubbles in mixture. The air pressure reached around 6 × 10^−2^ Pa. Uncured mixture was poured into the mould, and bubbles were eliminated again. (3) The mould with uncured mixture was put onto a thermal plate at 120 °C for half an hour.

**Figure 2:**
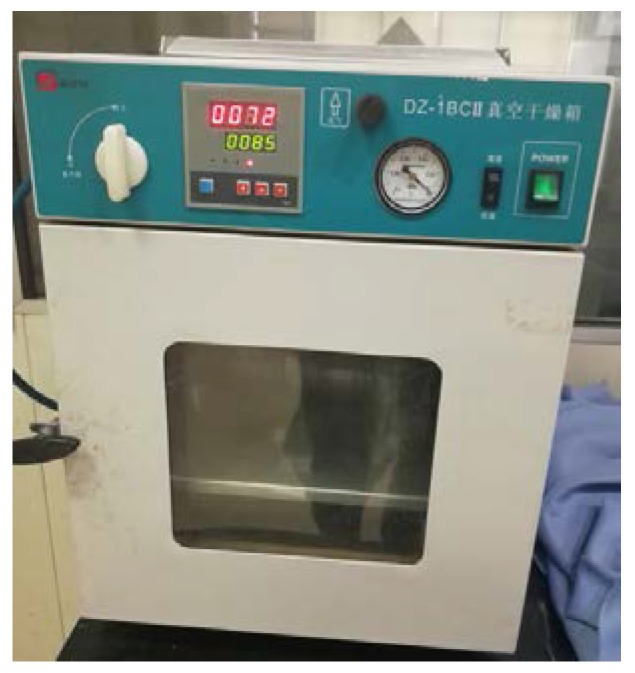
DZ-1BCII vacuum-drying oven.

An aging test of the silicone is carried out in this study; some groups’ samples are put into the vacuum-drying (TAISITE Instrument, Tianjin, China) oven shown in Figure 2 to exclude the influence of moisture and to obtain a purer composite. The temperature of the oven is set at 85 °C, and the aging times are controlled at 3, 6, 12 and 24 h for comparison.

**Figure 3:**
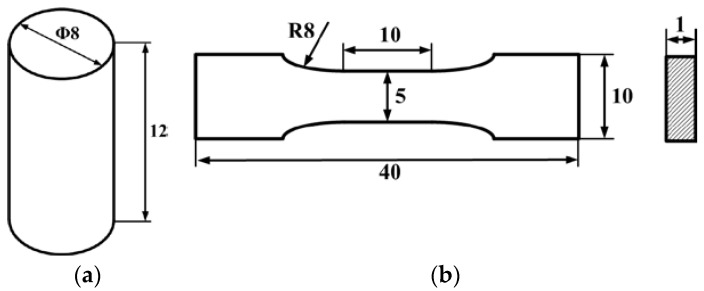
Dimension of the samples for experiment: (**a**) samples for compression test; (**b**) samples for tensile test.

The samples used for the compression test are shown in Figure 3a. The diameter of the cylindrical specimens is 8 mm, and the height is controlled at 12 mm. Because the height of silicone depends on the operation while filling the mould, all the samples are measured before test to guarantee the accuracy of strain calculation.

**Figure 4:**
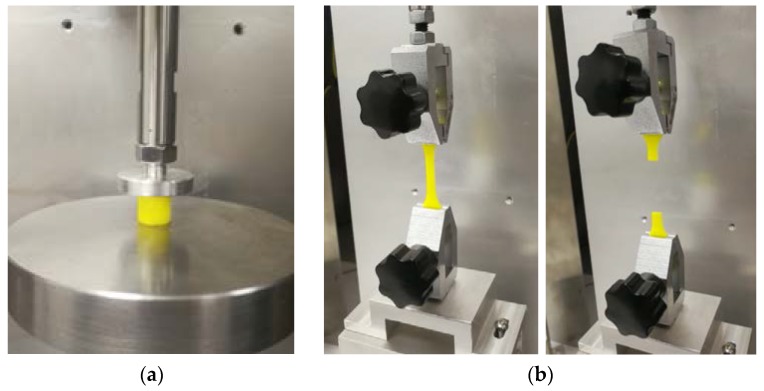
Process of material test: (**a**) Compression test; (**b**) Fracture of sample after tensile test.

To study the phosphors/silicone composite’s tensile strength and observe the influence of the mass fraction in the material’s tensile strength, tensile testing is necessary. A diagram of the sample is shown in Figure 3. Three groups of samples are prepared for tensile test, their mass fractions of phosphor are 0%, 20% and 40%. The tensile strain rate is controlled at 1.8 × 10^−3^/s. Figure 4 shows a standard fracture case of the tensile test, the sample just cracks at the middle position, which bears the maximum tensile stress. Both sides of the samples are glued to the fixture to avoid slippage during the tensile test. A voice coil motor (LCA50 series, manufactured by SMAC, Carlsbad, CA, USA) is used to obtain the reacting force and displacement data for calculating the stress and strain of silicone in all mechanical tests.

## 3. Numerical Simulation

**Figure 5:**
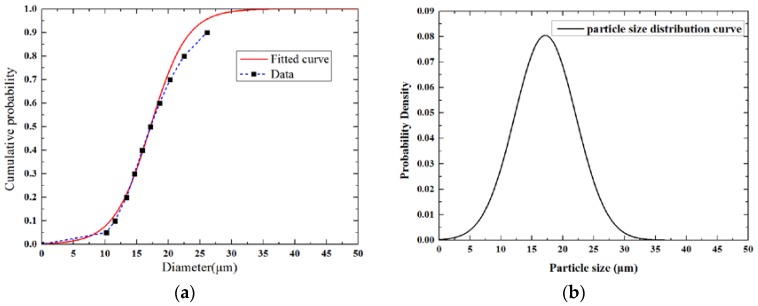
Raw data shown in Table 2 and fitted curve: (**a**) Cumulative density curve; (**b**) Probability density distribution curve.

The diameters of phosphor power are obtained by particle size distribution analysis (Grirem Advanced Materials, Beijing, China). The results of the cumulative probability of particles versus corresponding diameters are listed in Table 2. In general, the diameters of particles follow normal distribution. Thus, the data in Table 2 are used to extract the standard deviation and median diameter of the particles by Equation (1) [15].

(1) $$F(x)=\frac{\exp(a•(x-\mu ))}{1+\exp(a•(x-\mu ))},a=\frac{4}{\sqrt{2\pi }\sigma }$$

where *x* is the diameter of the particle, μ is the median diameter, σ is the standard deviation and *F*(*x*) is the cumulative probability where the diameter is less than value *x*. The standard deviation value is 4.956 μm, and the median diameter is 17.192 μm. Figure 5a shows that the fitted curve is highly consistent with the data obtained by particle size distribution analysis. Max error occurs when the cumulative probability reached 0.9, and the value of this error is below 8%. The Monte Carlo method is used to generate the diameters of the particles. Random dots are generated below the probability density distribution curve shown in Figure 5b. The values of the *x*-coordinate mean that the diameters of the particles are controlled within 50 μm to avoid extreme case. The simulation model is a cube with a side length of 80 μm. Phosphor particles are randomly distributed in this cube, and the mass fraction of particles is calculated until the required value is reached.

**Figure 6:**
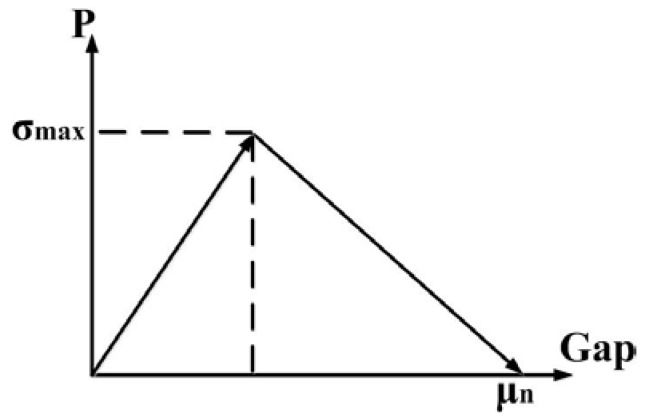
Normal contact stress and contact gap curve.

The coordinates of the particles are generated randomly within the range of a cube whose length is 80 μm. Contact elements are built up between phosphor particle and silicone. The adhesion behavior between particles and silicone is described by a cohesive zone material, which is based on the model proposed by Alfano and Crisfield [16]. It’s assumed that the separation mode of the interface surface normal to the interface dominates the slip tangent to the interface. The normal contact stress and contact gap behavior are plotted in Figure 6. The area value below the lines is equal to fracture energy. When the tensile stress reaches the value σ_max_, debonding of the interface starts, until the contact gap runs up to μ_n_. The value of max normal stress σ_max_ is set at 0.3 MPa, and the ultimate distance of the gap μ_n_ is 0.5 μm.

## 4. Discussion

### 4.1. Experimental Results

#### 4.1.1. SEM Observation

**Figure 7:**
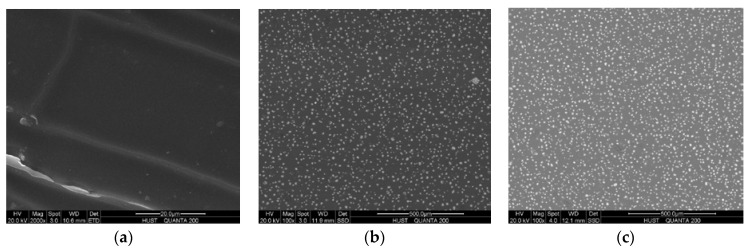
SEM skin layer of silicone composites with different mass fractions of phosphor: (**a**) 0%; (**b**) 20%; (**c**) 40%.

To ensure the uniformity of particles, samples are observed by scanning electron microscopy (SEM) after cooling down. The particles embedded in the silicone matrix shown in Figure 7 vary greatly in diameter. There are numerous tiny cracks distributed in the composite that could cause damage to the material. These flaws, which weaken the mechanical behavior, emerged during the curing and cooling stage. Cracks occur when uncured silicone in mould cools down, when the sample’s surface temperature changes faster than inside. This leads to the cracks mainly being distributed at the surfaces of samples, instead of inside the materials. To simplify the model, the impacts of flaws are neglected in this study.

#### 4.1.2. Mechanical Test Results

**Figure 8:**
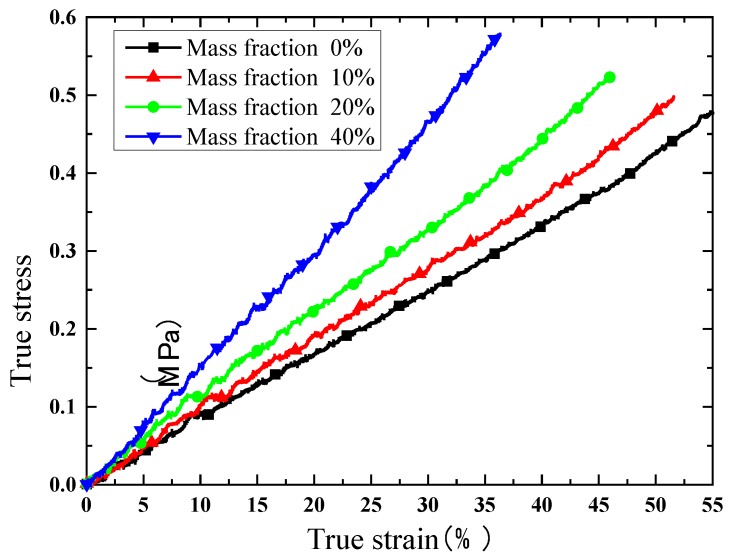
True strain versus stress curves at different mass fractions of phosphor with a strain rate of 8 × 10^−4^ s^−1^ and no aging.

**Figure 9:**
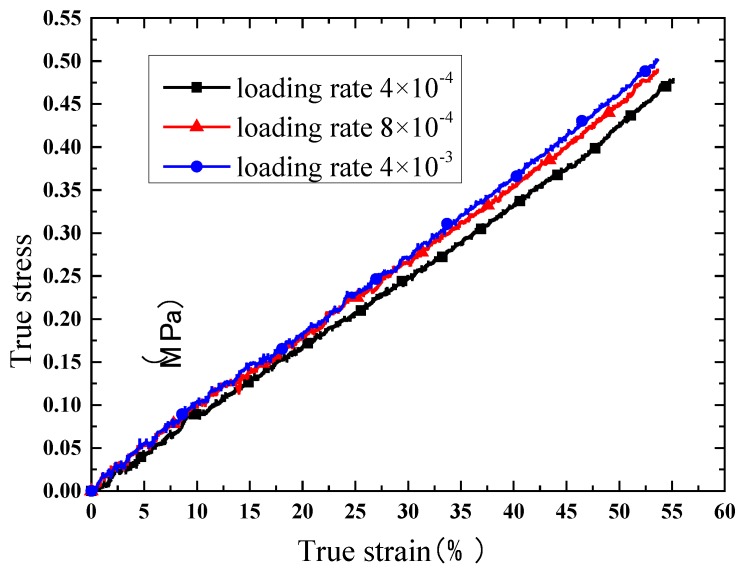
True strain versus stress curves of pure silicone at different loading rates (aging time: 0 h, mass fraction: 0%).

**Figure 10:**
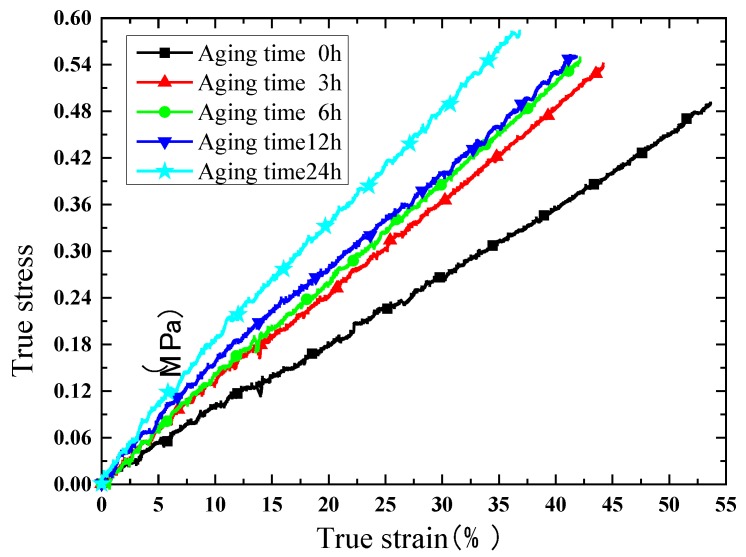
True strain versus stress curves at different aging times (strain rate: 8 × 10^−4^/s, mass fraction: 0%).

**Figure 11:**
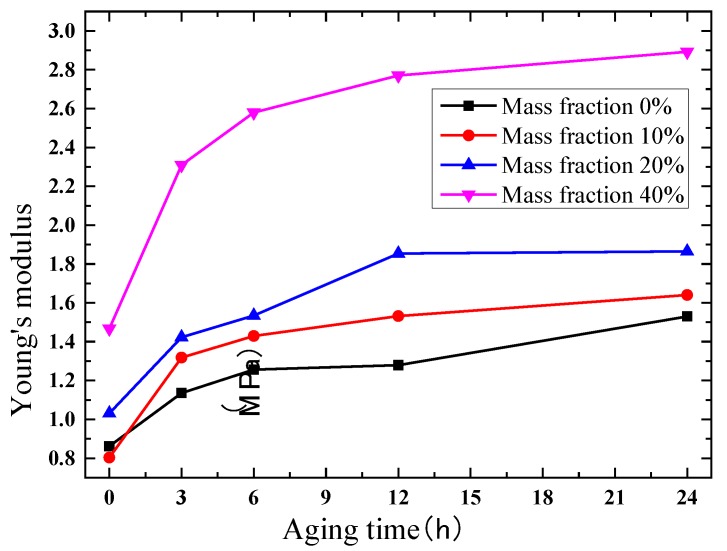
Elastic modulus of silicone under different conditions (strain rate: 8 × 10^−4^/s).

Due to the satisfactory elongation characteristic, true stress and true strain curves are extracted for comparison. The results of the compression test are shown in Figure 8, Figure 9, Figure 10 and Figure 11. Figure 8 indicates that the phosphors in the silicone stiffen the silicone matrix significantly. This is caused by the stiff particles embedded in the silicone. The properties of pure silicone at different loading rates are shown in Figure 9; the pure silicone material exhibits a slightly rate-dependent characteristic, which can be neglected below a strain rate of 4 × 10^−3^ s^−1^. The slope of the line at a loading rate of 4 × 10^−3^ s^−1^ is slightly larger than those at lower loading rates. Aging time has a great influence on the performance of the pure silicone; as shown in Figure 10, the Young’s modulus of the silicone increases significantly with aging time. This property of silicone could cause severe reliability issues in actual application, such as serious stress conditions and cracks in the material. The Young’s modulus of phosphor/silicone composite at strain rate of 8 × 10^−4^ s^−1^ is clearly shown in Figure 11.

**Figure 12:**
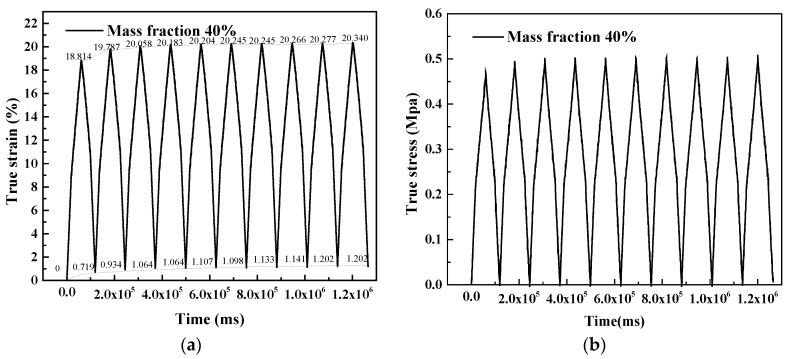
True strain and stress versus time in load-unload test: (**a**) Strain versus time; (**b**) Stress versus time.

**Figure 13:**
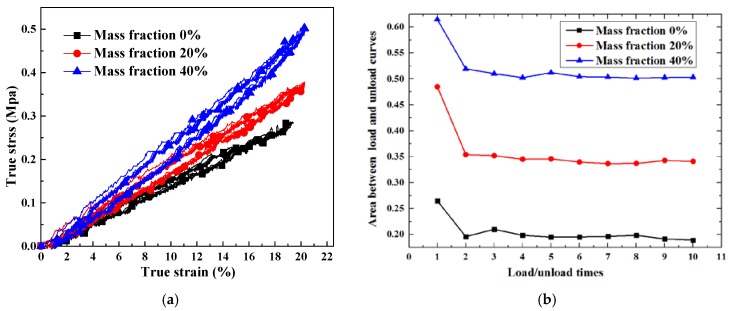
Load-unload test with different mass fractions of phosphor: (**a**) True strain versus true stress; (**b**) Areas between the load and unload curves versus load/unload times.

Figure 12 shows the measured strain rate for 4 × 10^−3^ /s and 20 °C room temperature for the samples whose mass fraction was controlled at 40%. The voice coil motor carries out a return motion when zero force is detected. It can be seen that the true strain is unable to return to zero, which means plastic strain has occurred. The energy stored in the composite has been consumed by the compression test. In Figure 13, ten compression cycles are applied to the samples, indicating the viscoelasticity of the silicone; this type of material will absorb the strain energy, and its stress will postpone strain. Figure 13b shows the values of areas between the load and unload curves versus load/unload times. After the first load and unload, the areas drop obviously to a stable level. This phenomenon can be explained by the debonding of the interface between particle and silicone matrix. Once debonding behavior occurs, it is barely able to return to the initial state. In the simulation, the forces between the interfaces are taken into consideration. Thus, it can be concluded that the material presents a progressive accumulation of plastic deformation.

**Figure 14:**
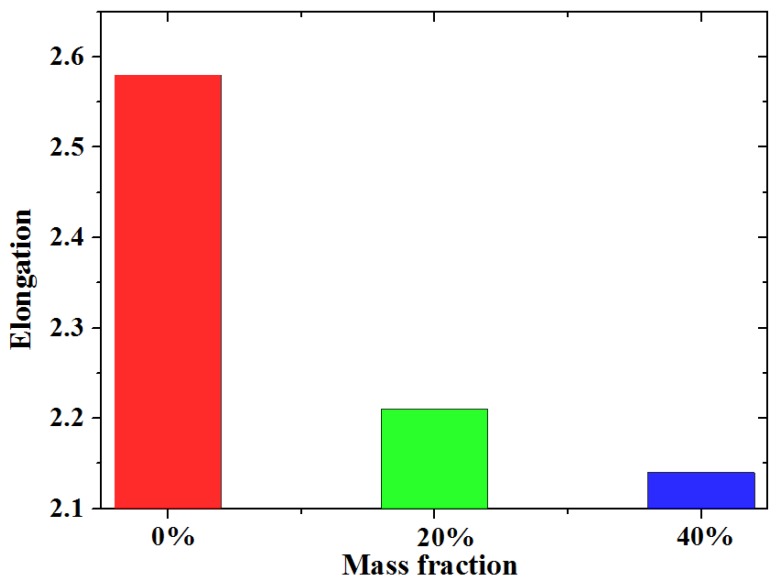
Elongation of the silicone with different mass fractions by experimental results (aging time 0 h, strain rate 1.8 × 10^−3^/s).

Figure 14 shows the results of the tensile test at a strain rate of 1.8 × 10^−3^/s. According to the tensile test, the elongation of the silicone decreases with an increasing mass fraction of phosphor. This means that phosphor distributed in silicone weakens the ductility of this composite. Maximum tensile stress when the mass fraction of phosphor is 0%, 20% and 40% is 1.61, 1.41, and 2.12 MPa, respectively. As the mass fraction of phosphors increases from 0% to 20%, the maximum tensile stress decreases 0.2 MPa instead. This proves that the phosphor weakens the samples. When the mass fraction reaches 40%, maximum stress rises to 2.12 MPa; this is mainly caused by the high Young’s modulus.

### 4.2. Numerical Simulation Results

**Figure 15:**
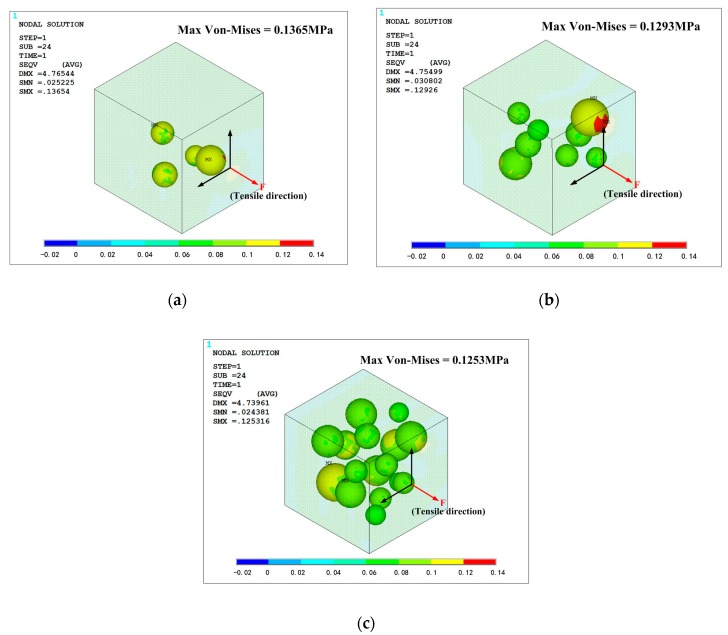
Von-Mises stress distribution of the phosphors/silicone composite: (**a**) mass fraction of 10%; (**b**) mass fraction of 20%; (**c**) mass fraction of 40%.

**Figure 16:**
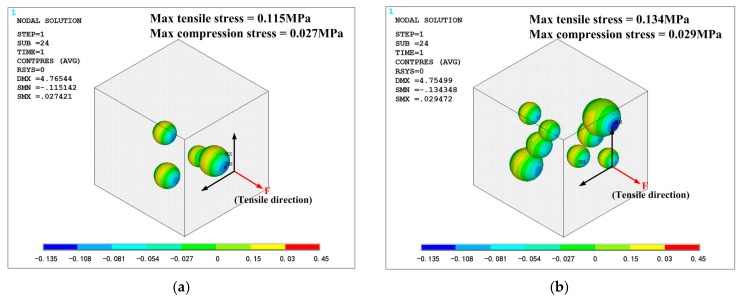

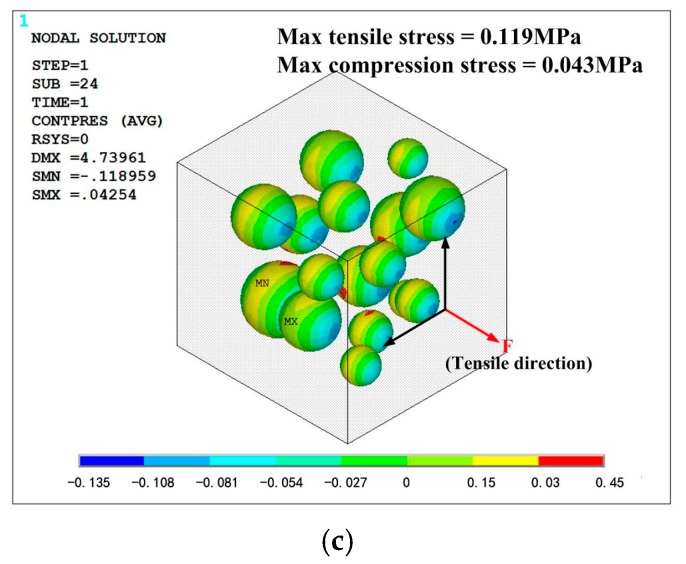
Contact stress between particles and silicone: (**a**) mass fraction of 10%; (**b**) mass fraction of 20%; (**c**) mass fraction of 40%.

All the simulation models are in same loading condition for comparing the effect of mass fraction. Tensile direction is shown in Figure 15 and Figure 16, and strain is controlled at 0.1 to avoid significant deformation of grid, which could cause difficulties in convergence. The opposite side of the cell is fixed in all direction. The Von-Mises stress distribution of the phosphors/silicone composite is shown in Figure 15; it can be seen that maximum stress decreases with the increase of mass fraction because fewer particles lead to stress concentration in the silicone matrix. When the number of particles increases, the composite becomes more uniform. As to the phenomenon of weak tensile performance, a reasonable explanation is that greater interface between particles and silicone causes defects in structural integrity. This result is consistent with the experimental observation based on the tensile test. Additionally, the maximum stress always occurs on the largest particle’s surface perpendicular to the extruded direction. When the tensile stress reaches a certain value, debonding behavior—which means a separation between the particles and the silicone matrix—will occur. Figure 15 shows the contact pressure; a negative value means that the interface between the particle and the silicone matrix experiences tensile stress. On the contrary, a positive value indicates that the interface experiences compression stress. As shown in Figure 16, the surfaces of the particles perpendicular to the extruded direction experiences tensile stress; meanwhile, the loop surface, which is the red region shown in Figure 16, experiences compression stress. Tensile stress, which could cause debonding of the interface, is 0.115, 0.134 and 0.118 MPa, respectively, for the corresponding mass fractions of phosphor of 10%, 20% and 40%, when the material’s engineering strain reached 10%. The stress condition on the interface gets worse when the mass fraction of the phosphor increases; as a result, elongation of the composite is weakened, which is consistent with the experiment shown in Figure 14.

## 5. Conclusions

Macro-scale mechanical behaviors of the phosphor/silicone composite are observed by compression and tensile testing. To explain the failure mechanism at the micro level, the finite element method was used with a cohesive zone material (CZM) model to express the contact state between silicone and phosphor. The experimental results of compression testing revealed that high mass fractions of phosphor, long aging times, and faster loading rates will stiffen the material of silicone sorted by extent of influence. The results of tensile testing indicated that the elongation properties decrease with an increase of phosphor mass fraction. According to the simulation, it was found that the size of the particles has a great influence on silicone’s tensile capability. Large differences in particle diameter will cause stress concentration at the interface of the largest one normal to the tensile direction. Due to the poor cohesion at the interface, high mass fractions may cause extensive fracture inside the silicone matrix. In conclusion, lower mass fractions of phosphor, uniform particle diameter, and good anti-aging properties are recommended for phosphor/silicone composites applied in LED packaging.

## Figures and Tables

**Table 1:** Mechanical properties of the materials.

Material	Density (g/cm^3^)	Young’s Modulus	Poisson’s Patio
Silicone	1.15	3.1	0.48
Phosphor	4.88	335,000	0.28

**Table 2:** Cumulative probability of particles versus diameters.

Cumulative probability	Diameter (μm)	Cumulative probability	Diameter (μm)
5%	≤10.1933	60%	≤18.6325
10%	≤11.5801	70%	≤20.2879
20%	≤13.3915	80%	≤22.4957
30%	≤14.6582	90%	≤26.1263
40%	≤15.9145		
